# Effect of Deep Cryogenic Treatment on Aging Strength of Mg–Al–Ca–Mn Alloy

**DOI:** 10.3390/ma18204769

**Published:** 2025-10-17

**Authors:** Mohamed Fouad, Taiki Nakata, Chao Xu, Jing Zuo, Zelin Wu, Lin Geng

**Affiliations:** 1 State Key Laboratory of Precision Welding and Joining of Materials and Structures, Harbin Institute of Technology, Harbin 150001, Chinagenglin@hit.edu.cn (L.G.); 2 Mechanical Engineering Department, Faculty of Engineering at Shoubra, Benha University, Cairo 11672, Egypt; 3 Mechanical Engineering Department, Nagaoka University of Technology, 1603-1 Kamitomioka, Nagaoka 940-2188, Japan

**Keywords:** Mg–Al–Ca–Mn alloy, solution treatment, deep cryogenic treatment, aging treatment, mechanical properties, microstructure characterization

## Abstract

T6 aging, involving solution treatment and artificial aging, is a widely adopted strengthening method for magnesium alloys due to its proven effectiveness. However, the integration of three or more sequential thermal treatments has been explored only sparingly, primarily due to the challenges associated with optimizing such multi-parameter processing systems. This study demonstrates that integrating a 12 h deep cryogenic treatment (DCT) before aging in a Mg–Al–Ca–Mn alloy optimizes mechanical performance, achieving a tensile strength of 343 MPa and 27.3% elongation. Microstructural analysis, based on electron backscatter diffraction (EBSD) and transmission electron microscopy (TEM), reveals that the strength enhancement results from ~29 nm precipitate refinement, elevated dislocation density, and nanoscale sub-grain formation, while the ductility gains stem from the activation of non-basal slip systems and the suppression of microcrack propagation. These synergistic mechanisms enable superior strain accommodation, providing a clear framework for DCT-enabled sequential heat treatment design in high-performance magnesium alloys.

## 1. Introduction

Mg alloys have emerged as attractive candidates for lightweight structural applications in automotive, aerospace, electronics, and defense industries. Their appeal stems from the combination of low weight, competitive mechanical performance, and excellent electromagnetic shielding, making them strong alternatives to rare-earth-based alloys [1]. Despite these advantages, their application remains limited due to poor formability and restricted ductility at room temperature, which arises from the limited number of active slip systems in their hexagonal close-packed crystal structure [2]. To address these shortcomings, several strengthening approaches have been explored, such as precipitation hardening [3,4], solid solution alloying [5], and grain refinement [6]. Another effective route involves weakening the strong basal texture typical of wrought Mg alloys, often achieved through rare-earth additions, though these raise material costs [7,8]. More economic alternatives, including alloying with Zr, Sr, or Ca, offer a cost-effective means of enhancing ductility [9]. Among various candidate systems, Mg–Al–Ca–Mn (AXM) alloys have recently gained attention as promising wrought materials due to their attractive property profile, including economical production, high strength, good ductility, corrosion resistance, and castability.

Sequential heat treatment techniques have been widely adopted as effective strategies for enhancing the mechanical performance of structural alloys in high-performance applications. Among these, the T6 aging process, comprising solution treatment followed by artificial aging, represents one of the most established and efficient strengthening routes. Its effectiveness primarily arises from the controlled precipitation of fine, coherent phases that impede dislocation motion, thereby markedly improving strength and hardness through precipitation hardening and dislocation–precipitate interactions [10,11,12,13,14]. In recent years, deep cryogenic treatment (DCT) has attracted considerable attention as a complementary process capable of further refining the microstructure and enhancing mechanical stability [15,16,17,18,19,20]. The DCT process promotes lattice contraction, dislocation multiplication, and solute redistribution, which collectively generate additional nucleation sites for fine precipitates and contribute to improved structural uniformity and stability. Despite the demonstrated benefits of T6 and DCT individually, their combined application has been scarcely investigated, primarily due to the difficulties of optimizing such complex, multi-parameter systems.

Recent studies [21,22,23] have shown that sequential heat treatment contributes significantly to microstructural refinement by promoting the formation of finely precipitates with uniform dispersed and reducing grain size and activation of non-basal slip systems. These modifications directly enhance strength, hardness, and ductility by influencing precipitation behavior and dislocation dynamics and deformation modes activation. The integration of multiple thermal treatments therefore enables precise tailoring of alloy performance to meet specialized industrial demands. Nevertheless, the mechanisms governing DCT in magnesium alloys remain insufficiently understood, which has hindered optimization of processing parameters.

In this context, the present study systematically examines the effect of DCT on the microstructural evolution and mechanical properties of T6-treated AXM alloy sheets. By varying DCT duration prior to artificial aging, the investigation employs advanced characterization techniques, including electron backscatter diffraction (EBSD) and transmission electron microscopy (TEM), to evaluate microstructural development and precipitation behavior. This approach provides new insights into DCT-enabled strengthening sequential pathways, establishing a framework for the design of AXM alloys with superior strength–ductility balance tailored for advanced engineering applications.

## 2. Experimental Procedure

### 2.1. Alloy Preparation and Heat Treatment

The AXM080202 alloy (Mg–0.82Al–0.18Ca–0.22Mn, wt.%) was designed to achieve an optimal balance between strength and ductility. Calcium promotes the formation of thermally stable Al_2_Ca and Ca_2_ (Mg, Al) phases, acting as grain-boundary pinning particles that inhibit grain growth and enhance high-temperature strength. Meanwhile, manganese refines the grain structure by reacting with impurities, while aluminum contributes to solid-solution and precipitation strengthening. The low alloying contents were selected based on previous studies [24,25], to ensure mechanical synergy, formability, and cost efficiency.

**Figure 1:**
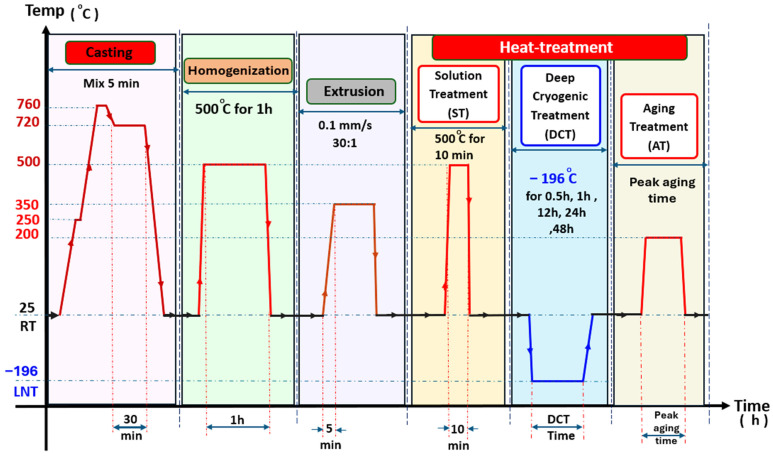
Schematic temperature–time profile for the AXM080202 alloy showing the sequential stages of casting, homogenization, extrusion, solution treatment (ST), deep cryogenic treatment (DCT), and aging (AT). Each step was designed to control solute distribution, phase stability, and precipitation behavior.

Each stage in the multi-step temperature–time profile (Figure 1) was precisely controlled to regulate solute distribution, refine grains, and stabilize precipitates, thereby enhancing the alloy’s mechanical properties. Cylindrical ingots (70 mm in diameter and 200 mm in height) were prepared by direct chill casting using an electric resistance furnace under a protective CO_2_–SF_6_ atmosphere to avoid oxidation. Pure Mg was melted at 760 °C, and then the Al–Ca–Mn master alloys were added at 720 °C, followed by mechanical stirring for 5 min to achieve compositional uniformity, and then a water-quenching solidification process. The chemical composition of the ingots was analyzed using inductively coupled plasma atomic emission spectroscopy (ICP-AES, ICAP 7000 series, Thermo Fisher Scientific, USA). Approximately 0.1 g specimens were extracted from the central cross-section of the ingot, and each measurement was repeated three times to ensure accuracy and reproducibility. The measured compositions were found to be in close agreement with the designed values, as depicted in Table 1.

**Figure 2:**
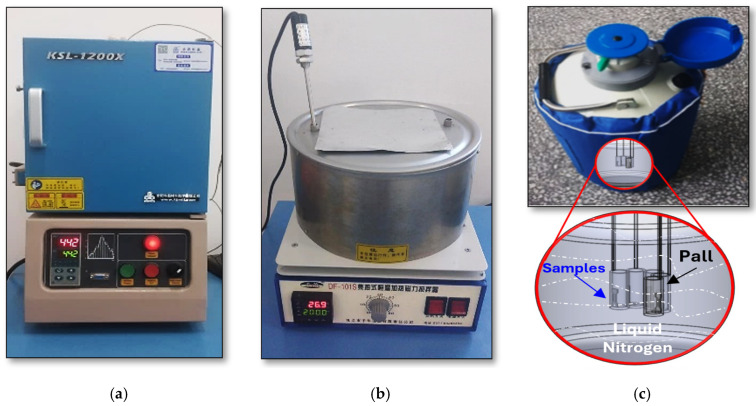
Heat treatment devices and equipment used, (**a**) electric furnace (KSL-1200X), (**b**) Yuhua DF-101S thermostatically controlled heating mantle with magnetic stirring, and (**c**) liquid nitrogen container, accompanied by a schematic illustrating sample placement during cryogenic treatment.

Ingots were homogenized at 500 °C for 1 h under a protected argon atmosphere in a OTF-1200X furnace (MTI Corporation, Zhengzhou, China), followed by water quenching, to dissolve secondary phases within the α-Mg matrix, and reduce resistance to thermomechanical processing. The homogenized ingots were machined into Ø52 mm × 60 mm billets according to the necessary volume for the final sheet geometry (40 mm × 4 mm) and then extruded at 350 °C through an indirect extrusion process with a speed of 0.1 mm·s^−1^, to form fine dynamically recrystallized grains. The extruded sheets subsequently underwent solution treatment (ST) at 500 °C for 10 min in a KSL-1200X electric furnace (MTI Corporation, Zhengzhou, China) (Figure 2a), followed by water quenching, to generate a supersaturated solid solution. Deep cryogenic treatment was then applied by immersing the solution-treated samples in the liquid nitrogen container at −196 °C for different soaking durations of 0.5–48 h (Figure 2c), to induce lattice strain and increased dislocation density, thereby providing additional nucleation sites for the formation of fine precipitates [26]. Finally, artificial aging (AT) was conducted at 200 °C in a Yuhua DF-101S heating mantle (Yuhua Instrument Co., Ltd., Shanghai, China) (Figure 2b) to achieve peak hardness and uniform precipitation strengthening throughout the alloy matrix, forming the SCAT samples group.

### 2.2. Mechanical Testing and Microstructural Characterization

#### 2.2.1. Mechanical Testing

**Figure 3:**
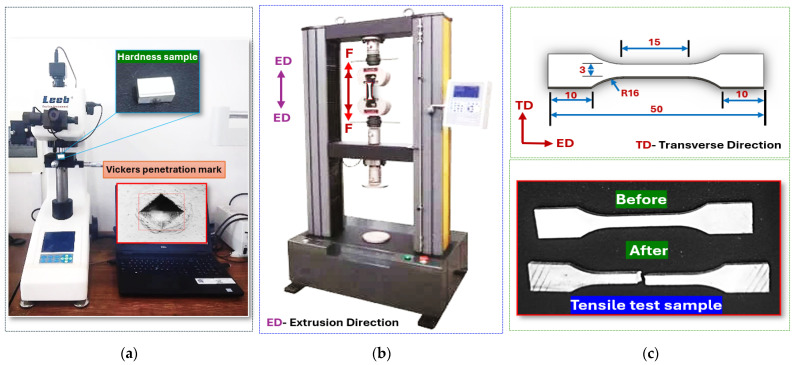
Material performance testing machines include (**a**) Huayin microhardness tester, (**b**) AG-X Plus Shimadzu Universal Testing Machine 20 kN, and (**c**) tensile specimens before and after the test.

The mechanical behavior of SAT and SCAT specimens was evaluated at room temperature through uniaxial tensile testing using an AG-X Plus universal testing machine (Shimadzu Corporation, Kyoto, Japan). Specimens were aligned along the extrusion direction (ED), and tests were carried out at a constant crosshead speed of 1 mm/min. Strain measurements were recorded with a 15 mm gauge length extensometer, as shown in Figure 3. Simultaneously, microhardness measurements were obtained using a Huayin tester (Huayin Testing Instrument Co., Ltd., Laizhou, China) under a 50 gf load and a dwell time of 15 s. Each hardness value represents the average of eight indentations, while tensile experiments were repeated three times for every treatment condition to confirm reproducibility.

#### 2.2.2. Microstructural Characterization

Microstructural and crystallographic texture analysis was performed on the ED–ND plane using a TESCAN scanning electron microscope )TESCAN ORSAY HOLDING, a.s., Brno, Czech Republic) equipped with a TSL electron backscatter diffraction (EBSD) system. EBSD scans were acquired with a step size of 0.5 μm and data processed using EDAX-TSL OIM (version 8) and ATEX (version 4.12) software. Precipitation features were further characterized by transmission electron microscopy (FEI Talos F200X, FEI Company, Hillsboro, OR, USA), with secondary phases identified through high-angle annular dark-field (HAADF) imaging in STEM mode. Quantitative precipitate measurements were carried out using Image-Pro Plus software (version 10). Specimen preparation for EBSD involved sequential grinding with SiC abrasive papers up to 4000 grits, followed by polishing with a 0.05 μm OP-S suspension (Struers OP-S, Struers A/S, Ballerup, Denmark) to remove surface scratches. Final polishing was achieved electrolytically at −2 °C for 15 s in an electrolyte of 10% perchloric acid and 90% ethanol, using a current of 0.20 A and 20 V. Samples were then ultrasonically cleaned in methanol and air-dried prior to scanning. TEM foils, 3 mm in diameter and ~0.04 mm thick, were thinned using a GATAN695 ion milling system (Gatan, Inc., Pleasanton, CA, USA) operated at 4.3 kV and 40 μA. Initial milling was performed at an angle of 6°, followed by final thinning at 2° to ensure an electron-transparent region suitable for high-resolution imaging.

## 3. Results

### 3.1. Mechanical Properties and Microstructural Characterizations

#### 3.1.1. Mechanical Properties

**Figure 4:**
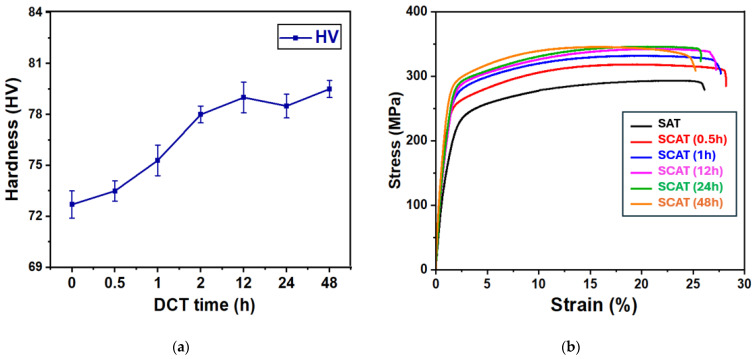
Mechanical properties of aging-treated AXM alloy sheets subjected to cryogenic treatment for durations of 0.5 h, 1 h, 12 h, 24 h, and 48 h: (**a**) hardness evolution and (**b**) tensile behavior.

The influence of DCT duration on the mechanical response of SAT-aged AXM080202 alloy sheets within the sequential cryogenic-aging treatment (SCAT) process is presented in Figure 4. A clear enhancement in mechanical properties was observed following the application of DCT. Hardness increased progressively with soaking time, reaching its maximum value of 79 HV after 12 h of treatment (Figure 4a). A similar trend was identified for strength, as both yield strength and ultimate tensile strength achieved peak average values of 275 MPa and 343 MPa, respectively, after 12 h of cryogenic exposure (Figure 4b). In contrast, ductility exhibited an inverse relationship with treatment duration. The highest elongation (28.2%) was recorded after 0.5 h of DCT, followed by a gradual decline at longer soaking periods. This nonlinear behavior highlights the critical role of optimizing cryogenic treatment duration to balance strength and ductility. Among all the conditions investigated, the 12 h DCT proved most effective, yielding increases of 21.7% in yield strength and 18.3% in ultimate tensile strength, while maintaining ductility at 27.3%, corresponding to a modest 5% improvement, as explained in detail in Table 2. The mechanisms underpinning these improvements are addressed in the following sections.

**Figure 5:**
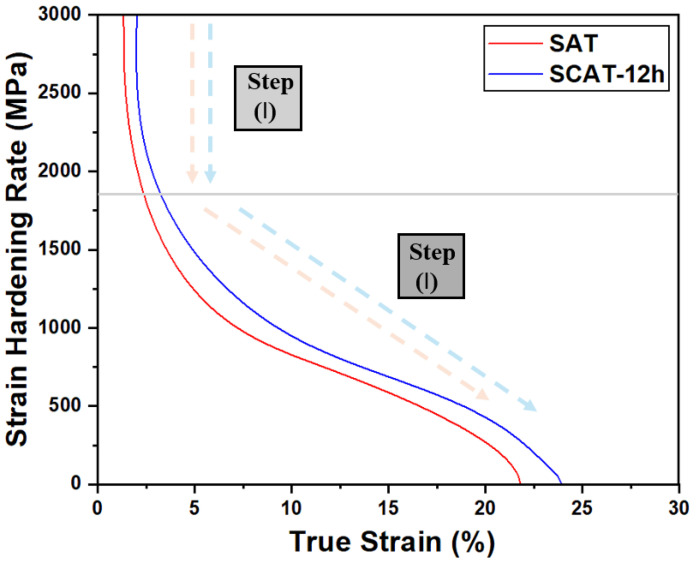
Strain hardening rates as functions of true strain for SAT and SDAT samples.

To assess the effect of deep cryogenic treatment on the strain hardening behavior of aging treated alloy, the macroscopic strain hardening rate (H) of SAT and SCAT (12 h) samples were determined using the ratio between change in the true stress (dσ) and true plastic strain (dε) [27,28]. As illustrated in Figure 5, the strain hardening rate exhibits a two-stage monotonic decline under both treatment conditions. In Stage I (ε < 2%), comparable hardening responses were observed, reflecting the transition from elastic to plastic deformation. The steep reduction in hardening rate at this stage is associated with the narrow elastoplastic regime [29,30]. In Stage II (ε ≥ 2%), a noticeable change in the slope of the hardening curve indicates a transition in the dominant deformation mechanisms, consistent with previous studies [28]. The SCAT (12 h) samples exhibited a delayed onset of necking, accompanied by improved tensile strength and ductility over an extended strain range. Specifically, the SCAT-12 h samples demonstrated superior strain hardening capacity, characterized by a higher hardening rate and a more uniform elongation than SAT sample.

#### 3.1.2. Microstructural Characterizations

**Figure 6:**
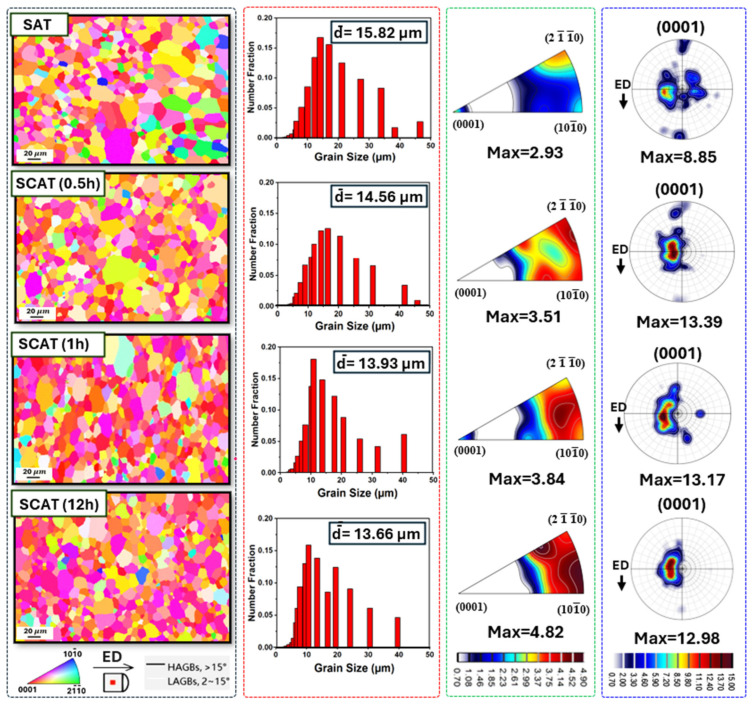
EBSD analysis of the aging-treated AXM alloy sheet subjected to cryogenic treatment at soaking durations of 0.5 h, 1 h, and 12 h, including IPF maps, grain size distribution diagrams, inverse pole figures, and pole figures.

EBSD analysis was employed to examine the microstructural evolution of aging-treated AXM080202 alloy sheets subjected to various DCT durations within the SCAT sequence. The inverse pole figure (IPF) maps presented in Figure 6 revealed a heterogeneous grain morphology, consisting of both fine and coarse grains. Pole figure (PF) analysis indicated that the basal texture, initially concentrated along the normal direction (ND), intensified progressively with increasing DCT duration. Simultaneously, IPF results demonstrated gradual grain reorientation, where grains tended to rotate preferentially between the $\langle 10\bar{1}0\rangle$ and $\langle 2\bar{1}\bar{1}0\rangle \;$ orientations. Importantly, no evidence of deformation twinning was observed in any of the investigated samples, suggesting that the sequence treatment does not initiate twinning. Beyond texture modification, DCT also promoted grain refinement. The average grain size decreased from 15.82 μm in the SAT condition to 13.66 μm after 12 h of cryogenic exposure. This reduction in grain size highlights the capacity of extended DCT to improve microstructural uniformity and enhance grain boundary strengthening, thereby contributing to improved mechanical performance of the alloy.

**Figure 7:**
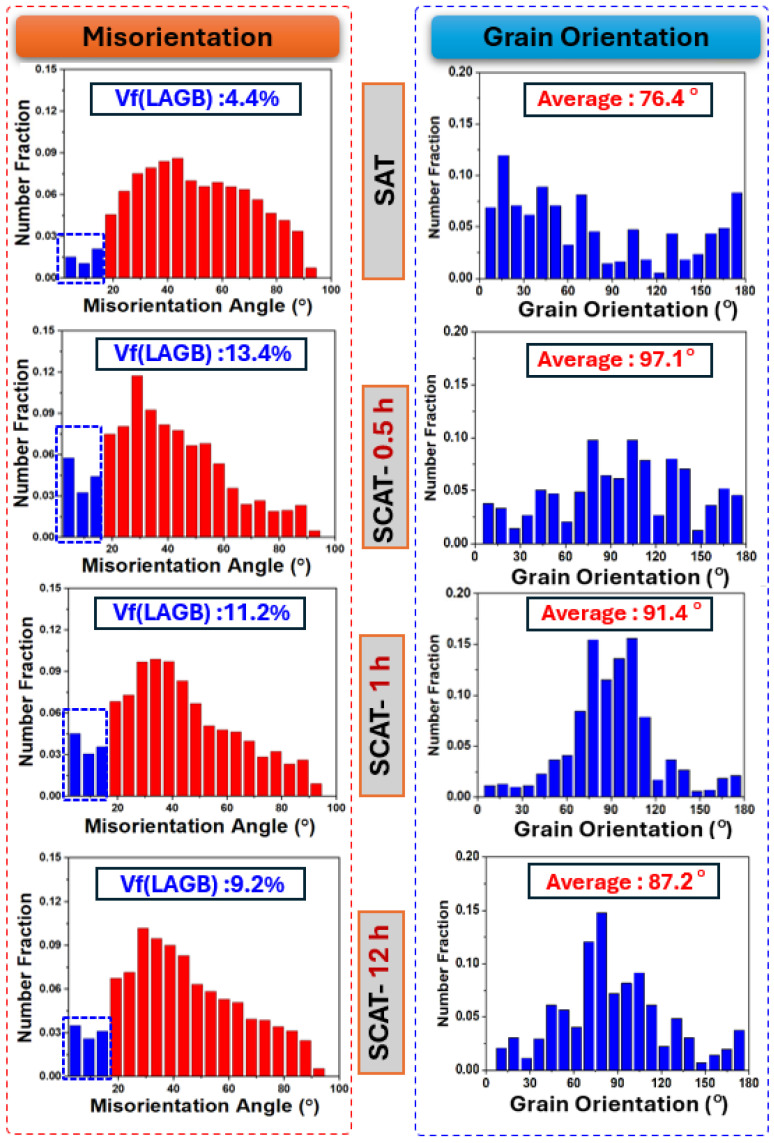
EBSD analysis of aging-treated AXM alloy sheets subjected to different soaking durations during the DCT process. Including, misorientation angle distribution and grain orientation distribution.

As illustrated in Figure 7, deep cryogenic treatment induces pronounced changes in grain boundary misorientation distributions. In the SAT condition, the fraction of low-angle grain boundaries (LAGBs, <15°) was measured at 4.4%. After 0.5 h of using DCT, this fraction increased to 13.4%, reflecting the formation of sub-grain structures driven by microstructural reorganization and accompanied by highest grain rotation by 97°, as shown in grain orientation analysis. With prolonged cryogenic exposure, the grain boundary configuration evolved further. After 12 h of DCT, the LAGB fraction decreased to 9.2%, suggesting that part of the LAGBs had transformed into high-angle grain boundaries (HAGBs) through dislocation annihilation [31]. This transformation contributed to enhanced grain stability and reduced grain rotation, consistent with the observations in grain orientation analysis. Taken together, these findings indicate that short-term DCT promotes LAGB formation, whereas extended DCT favors their conversion into HAGBs, highlighting the progressive evolution of grain boundaries under sustained cryogenic conditions.

**Figure 8:**
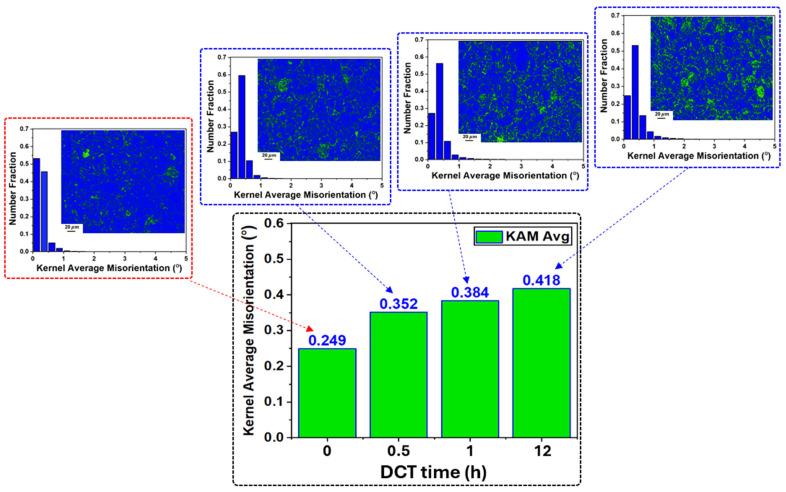
KAM analysis of aging-treated AXM alloy sheets subjected to cryogenic treatment for soaking durations of 0.5 h, 1 h, and 12 h, accompanied by the corresponding KAM distribution diagrams and KAM maps.

The evolution of dislocation density was assessed using kernel average misorientation (KAM) analysis obtained from EBSD data [32]. The geometrically necessary dislocation density ($\rho^{GND}$) are defined using the relation

(1) $$\rho^{GND}=\frac{2\varphi }{\mu b}$$

where φ represents the average KAM value, μ is the step size, and b is the Burgers vector. Since μ and b remain constant, changes in $\rho^{GND}$ are directly proportional to variations in φ. As shown in Figure 8, a progressive increase in dislocation density was observed with extended DCT duration, reaching 0.418° after 12 h compared with 0.249° in the SAT condition. This trend is reflected by the expansion of green regions in the corresponding KAM maps. Dislocations were predominantly concentrated near grain boundaries, where they restricted mobility and promoted intragranular entanglement. Elevated KAM values within coarse grains further indicated the presence of dense internal dislocation networks, consistent with the development of sub-grain structures [33,34]. These findings confirm that prolonged cryogenic soaking effectively enhances dislocation density.

**Figure 9:**
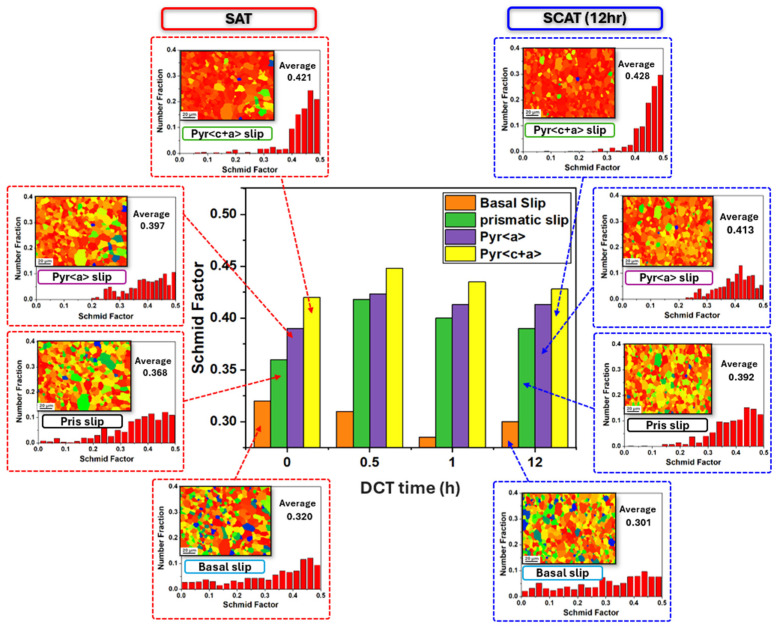
Schmid factor analysis of aging-treated AXM alloy sheets subjected to cryogenic treatment for soaking durations of 0.5 h, 1 h, and 12 h, accompanied by the corresponding SF distribution profiles and SF maps of SAT and SCAT (12 h) samples.

The influence of DCT on slip system activation was examined using Schmid factor (SF) analysis based on EBSD measurements [35,36]. A high SF value generally indicates a greater probability of slip system activation. As illustrated in Figure 9, addition of cryogenic treatment within sequential treatment reduces the activity of the basal slip system, as reflected by reduced SF values. By contrast, non-basal systems exhibited enhanced activation, particularly pyramidal ⟨c+a⟩ slip. The SF values of these systems reached their maximum after 0.5 h of using DCT and then declined slightly under longer soaking times. This behavior is attributed to grain rotations induced by cryogenic treatment, as also depicted in grain orientation analysis in Figure 7.

### 3.2. Precipitation Behavior

**Figure 10:**
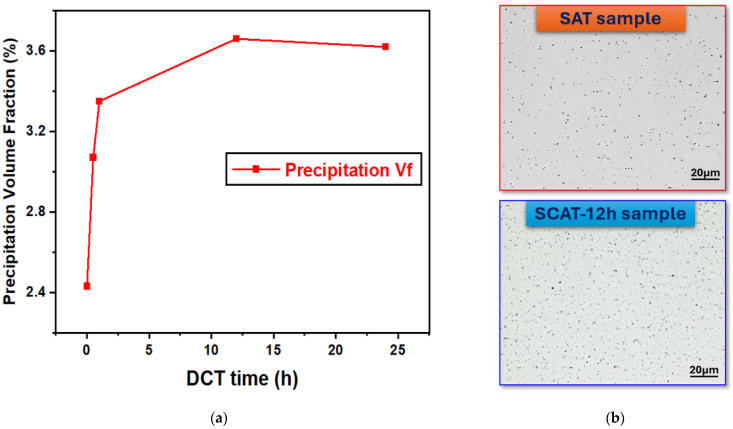
Volume fraction analysis of second-phase precipitates in AXM alloy sheets subjected to SAT and SCAT processes. (**a**) precipitation volume fraction diagram with using different DCT durations, (**b**) precipitation maps for SAT and SCAT (12 h) samples.

The effect of DCT duration on the precipitate volume fraction (Vf) of aging-strengthened AXM alloy sheets was investigated through quantitative analysis, as shown in Figure 10. During the early stages of treatment, Vf increased markedly and reached its maximum after 12 h of using DCT. At this stage, the precipitation maps revealed a uniform distribution of secondary phases. When the treatment was extended to 24 h, however, Vf decreased slightly, indicating that saturation had been reached and the thermodynamic driving force for further precipitation was reduced.

This behavior is consistent with the precipitation kinetics described by the Kolmogorov–Johnson–Mehl–Avrami (KJMA) model [37]:

(2) $$∆Vf=1-exp(kt^{n})$$

**Figure 11:**
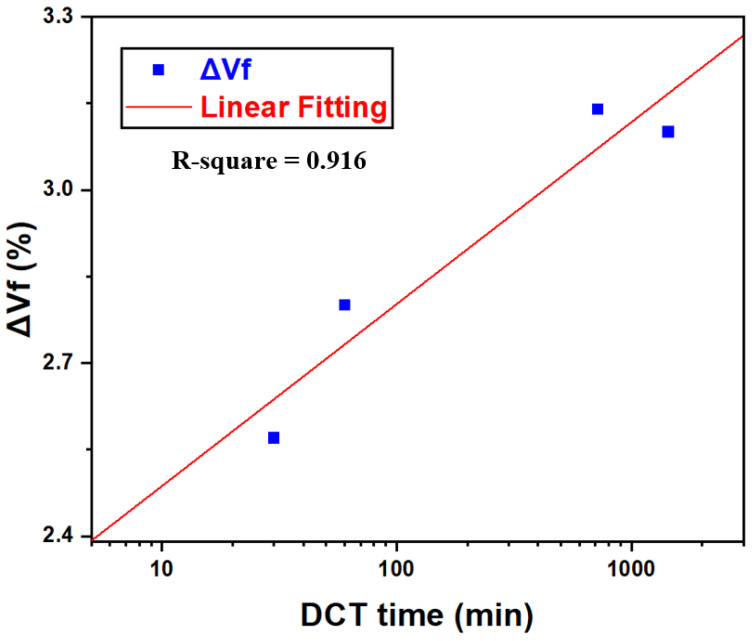
Increment in precipitate volume fraction of aging-treated AXM alloy sheets as a function of DCT duration.

In this relation, ΔVf denotes the increase in secondary-phase precipitates, k represents the kinetic constant, n is a temperature-independent exponent, and t corresponds to the treatment time. Within this framework, ΔVf reflects the incremental precipitation promoted by cryogenic soaking. Regression analysis of the experimental results yielded kinetic parameters of k = 0.0013 and n = 0.425. The variation in ΔVf as a function of DCT duration is presented in Figure 11.

**Figure 12:**
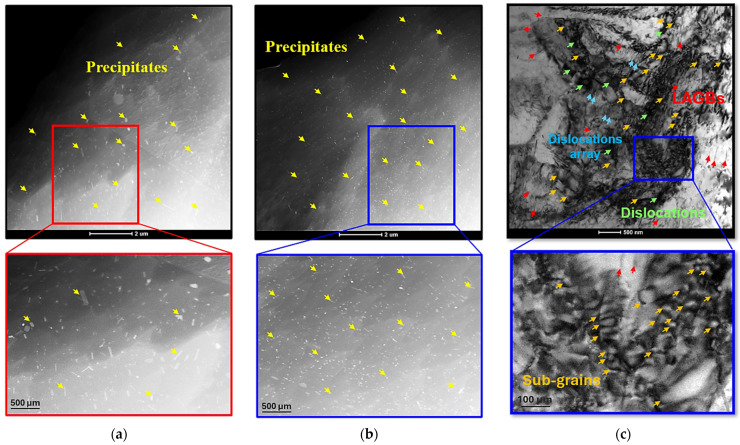
Typical bright-field TEM micrographs of the AXM080202 alloy sheet: (**a**) SAT sample and (**b**,**c**) SCAT sample with 12 h for DCT.

The introduction of deep cryogenic treatment into the thermal treatments sequence produced a pronounced modification in precipitate morphology, as illustrated in Figure 12a,b. Under the SAT condition, the microstructure was dominated by needle-shaped precipitates, whereas the SCAT-12 h specimen exhibited a finer and more homogeneous distribution of spherical precipitates. The average precipitate diameter decreased from 42 nm in SAT sample to 29 nm, accompanied by an increase in precipitate density. This refinement is attributed to the enhanced precipitation kinetics facilitated by DCT [23,38]. Specifically, immersion in liquid nitrogen induces lattice contraction, which increases the driving force for nucleation and promotes the formation of additional precipitates. Intragranular analysis of the SCAT specimen (Figure 12c) further revealed that dislocations (highlighted by green arrows) were reorganized into arrays and walls (indicated by blue arrows). The elevated dislocation density, resulting from cryogenic effects [20,26], subsequently promoted the development of low-angle grain boundaries (shown as red arrows) and nanoscale grains ranging from 30 to 120 nm (orange arrows), these modifications enhanced by aging treatment.

**Figure 13:**
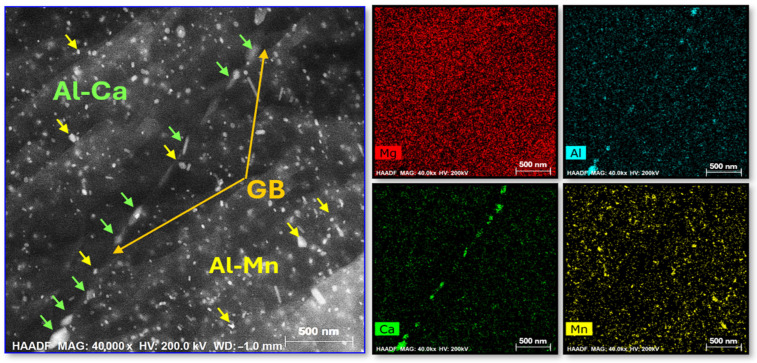
HAADF-STEM micrograph of the SCAT sample with 12 h for DCT, with corresponding EDS elemental maps for Al, Ca, and Mn at the grain boundary (GB).

High-angle annular dark-field (HAADF) imaging of the SCAT sample with 12 h for DCT (Figure 13) provided additional evidence for this microstructural evolution, revealing a dense distribution of intragranular nanoscale precipitates, in some cases exhibiting partial spatial overlap. Two distinct phases were identified: Al–Mn phase (indicated by yellow arrows) and Al–Ca phase (highlighted by green arrows). Beyond the intragranular regions, precipitates were also observed at grain boundaries. Consistent with earlier findings [26], these precipitates preferentially segregated at HAGBs rather than at LAGBs, owing to the lower thermodynamic driving force for segregation at small misorientation angles. Such boundary-associated precipitates function as effective pinning sites, restricting grain boundary mobility and thereby enhancing boundary strengthening. This mechanism contributes directly to the superior mechanical performance exhibited by the SCAT samples.

### 3.3. Fracture Characterization

**Figure 14:**
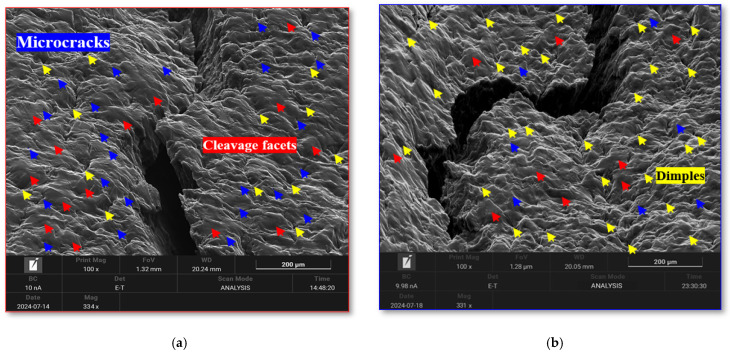
Fracture morphology characterization of the AXM080202 alloy sheet. (**a**) SAT case, (**b**) SCAT (12 h) case.

Fracture characterization offered additional insight into the effects of cryogenic treatment. SEM analysis of tensile fracture morphologies (Figure 14) revealed that the SCAT specimen exhibited a more tortuous fracture profile compared with the SAT specimen, indicating improved energy absorption before failure after using DCT. Both conditions contained cleavage facets (red arrows), ductile dimples (yellow arrows), and microcracks (blue arrows). However, the SAT specimen was distinguished by larger dimples, greater microcrack density, and extensive cleavage features, all of which are indicative of reduced ductility, consistent with prior reports [16,39]. In contrast, the SCAT specimen exhibited deeper, more uniformly distributed dimples with fewer microcracks, indicating a predominantly ductile fracture mode and enhanced strain hardening after using DCT.

## 4. Discussion

The duration of deep cryogenic treatment plays a decisive role in governing the microstructural evolution of aging-treated AXM080202 alloy sheets, influencing crystallographic texture, dislocation activity, and precipitation kinetics. As illustrated in Figure 4b, DCT introduces time-dependent improvements in both strength and ductility. Maximum elongation was achieved after 0.5 h of treatment, whereas peak tensile strength was attained following 48 h of DCT exposure. Notably, a balanced combination of mechanical properties was obtained at 12 h of DCT, with a strength increase of 18.3% and an elongation improvement of 5%, underscoring the importance of process optimization.

**Figure 15:**
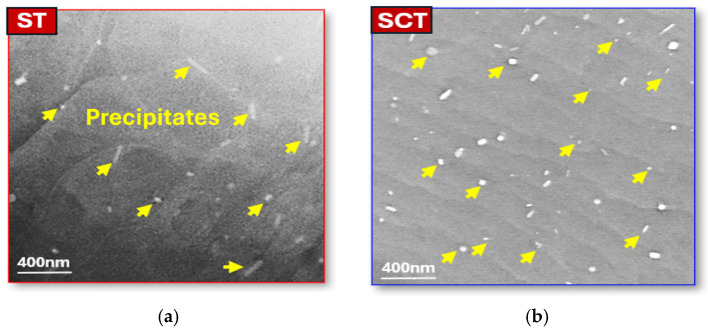

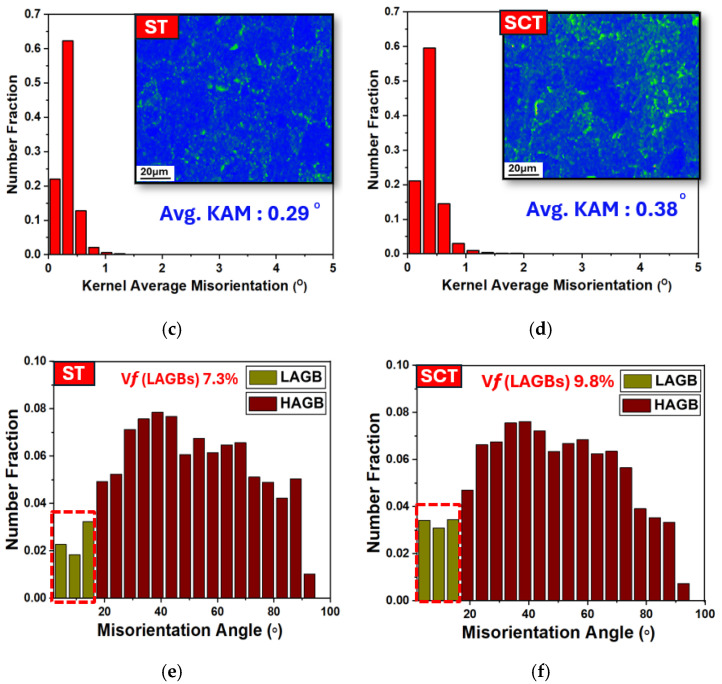
Influence of deep cryogenic treatment on the microstructure of a solution-treated AXM080202 alloy sheet: (**a**,**b**) precipitation characteristics analysis by bright-field TEM observations, (**c**,**d**) dislocation density analysis via KAM maps and distributions, (**e**,**f**) grain boundary character analysis from misorientation distributions.

To gain a deeper understanding of the DCT mechanism, the optimally treated SCT sample (12 h), which underwent solution treatment followed by cryogenic processing, was examined before aging and compared with the ST sample. As shown in Figure 15a,b, TEM analysis revealed distinct differences in precipitate morphology and distribution between the two conditions. The DCT-treated sample exhibited a higher volume fraction of precipitates with finer, uniformly dispersed spherical morphologies, indicating that DCT promotes a transformation from elongated to spherical precipitates, thereby improving both refinement and homogeneity. This enhancement can be attributed to the elevated vacancy concentration retained after solution treatment. Subsequent immersion in liquid nitrogen induces matrix contraction, leading to a reduction in vacancy density and promoting uniform precipitate nucleation through vacancy and solute atom redistribution. Simultaneously, compressive stresses generated during cryogenic exposure stimulate dislocation formation via vacancy annihilation mechanisms. KAM analysis (Figure 15c,d) confirmed this trend, showing a higher residual dislocation density in the SCT sample (0.38°) compared with the ST sample (0.29°). Furthermore, grain boundary misorientation analysis (Figure 15e,f) demonstrated that DCT increased the fraction of low-angle grain boundaries from 7.3% in the ST sample to 9.8% in the SCT sample, suggesting enhanced sub grain formation that impedes dislocation motion and leads to an additional enhancement of dislocation density (Figure 15e,f). Overall, the synergistic combination between precipitation and dislocation strengthening induced by DCT served as the primary mechanism responsible for the enhanced strength of the AXM alloy sheets.

At short DCT durations, highest grain rotation was observed (Figure 7), particularly along the $\langle 2\bar{1}\bar{1}0\rangle$ and $\langle 10\bar{1}0\rangle$ orientations (Figure 6). This behavior facilitated further activation of non-basal slip systems (Figure 9). Notably, the additional activation of pyramidal ⟨c+a⟩ slip systems further mitigated twinning-induced crack formation and improved strain compatibility along the c-axis [40], which enhanced plastic deformability by suppressing microcrack initiation during tensile loading. Microstructural analyses provided further evidence of these mechanisms. SEM observations (Figure 14) revealed that DCT-treated samples exhibited a reduced density of surface microcracks compared with SAT counterparts, which is also attributed to grain boundary stabilization through precipitate pinning (Figure 13). These effects collectively delayed fracture onset in DCT-treated samples (Figure 14), Which was reflected in more ductile fracture features of SCAT samples, improving energy absorption capacity and thus increasing strain hardening after using DCT (Figure 5). These mechanisms collectively account for improvements elongation reported in Figure 4b, its behavior is linked to the behavior of grain rotation and the activation of non-basal slip systems.

With increasing DCT time (≥0.5 h), elongation gradually decreased while strength improved, as depicted in Figure 4, following a two-stage trend characterized by a rapid rise within the first hour and a slower increase thereafter. This strengthening response is closely linked to precipitation kinetics, dislocation interactions, and grain refinement. For precipitation kinetics, TEM observations (Figure 12) revealed that DCT refined the precipitate morphology, producing smaller precipitates (29 nm vs. 42 nm in the SAT sample). Interestingly, DCT promotes precipitation by annihilating supersaturated vacancies retained from solution treatment, a result of cryogenic lattice contraction [23,38]. As depicted in Figure 10, precipitation behavior followed two stages, there first rapid nucleation in the early period, followed by saturation as the thermodynamic driving force diminished, in agreement with KJMA kinetics (Equation (2)) [27]. The strengthening effect of precipitates was primarily governed by the Orowan mechanism [32]:

(3) $$\sigma_{Orowan}=M\frac{0.4bG_{m}}{\pi d\left(\sqrt{1-\upsilon }\right)\left(\sqrt{\frac{\pi }{{4V}_{p}}}-1\right)}\ln\left(\frac{r}{b}\right)$$

where *M* is the Taylor factor, *v* is Poisson’s ratio, *b* is the Burgers vector, *G* is the shear modulus, *r* is the precipitate radius, and *Vp* is the precipitate volume fraction. Fine, dense precipitates in SCAT samples acted as strong obstacles to dislocation motion, thereby requiring higher stresses for bypass. In addition, grain boundary precipitates contributed to stability through Zener pinning [41]:

(4) $$P_{Z}=\frac{3}{4}\gamma \frac{f}{r}$$

where *P_Z_* is the pinning pressure, *γ* is the grain boundary energy, *f* is the precipitate volume fraction, and *r* is the particle radius.

For dislocation and boundary evolution, microstructural analysis showed that the fraction of LAGBs increased to 13.4% after 0.5 h of DCT (Figure 7). These boundaries initially acted as effective barriers to dislocation motion, increasing dislocation density (Figure 8). With prolonged exposure, LAGBs progressively transform into HAGBs due to the dislocation effect [21]. This contributed to the alloy strengthening through stabilizing grain boundaries. Despite this reduction in LAGB formation, dislocation density continued to increase, albeit at a reduced rate, indicating that while grain boundaries serve as dislocation storage sites, they are not the sole contributors to enhanced dislocation. The continuous increase in dislocation density with longer DCT durations is best explained by the combined effects of dislocations, LAGBs, and precipitates, which produced a two-stage strengthening response, as shown in Figure 4b, rapid hardening during the initial phase due to LAGB prevalence, enhanced dislocation and rapid precipitation, followed by a slower stage as boundary transformation into HAGB and precipitation saturation limited further strengthening. Dislocation–boundary interactions also facilitated the formation of Lomer–Cottrell locks [33], further impeding dislocation glide and enhancing strain hardening (Figure 5). Moreover, LAGBs contributed to precipitate shearing and redistribution, which increased deformation resistance [34].

For grain refinement, TEM analysis (Figure 12c) confirmed that DCT generated submicron grains in the range of 30–120 nm, contributing additional strengthening through grain boundary hardening. Furthermore, grain size decreased from 15.82 µm in SAT specimens to 13.62 µm in SCAT samples treated for 12 h, corresponding to a 14% reduction (Figure 6). This refinement, consistent with the Hall–Petch relation [42], resulted in an estimated yield strength improvement of ~21.7% (Figure 4). Finally, these findings provide conclusive evidence that cryogenic treatment, when integrated with solution and aging processes, exerts a decisive influence on enhancing the mechanical performance of AXM alloy sheets.

## 5. Conclusions

This study examined the influence of deep cryogenic treatment (DCT) duration on the microstructural evolution and mechanical performance of aging (T6)-strengthened AXM080202 alloy sheets. Microstructural characterization using SEM, EBSD, and TEM enabled a detailed assessment of microstructural evolution and precipitation behavior. The key findings can be summarized as follows:

1. Optimal Treatment Duration: A 12 h DCT through sequential treatment provided the most favorable mechanical property balance, yielding a peak hardness of 79 HV, tensile strength of 343 MPa (+18.3%), and elongation to failure of 27.3% (+5%). Longer durations enhanced strength but reduced ductility, highlighting the need for optimized cryogenic exposure time.
2. Synergistic Strengthening Mechanisms: Strength improvements arose from refined (~29 nm) precipitates uniformly distributed within the matrix, nanoscale grain formation (with a range of 30–120 nm), grain boundary strengthening, and elevated dislocation density, which collectively contributed to substantial hardening.
3. Ductility Enhancement Mechanisms: Improved ductility was linked to enhanced grain rotation, activation of non-basal slip systems, and suppression of premature microcrack initiation during deformation, enabling greater strain accommodation.

In conclusion, this work presents a systematic framework for elucidating the mechanisms of DCT-mediated strengthening in AXM alloys through sequential thermal treatments. The findings provide a solid foundation for the development of alloys with optimized strength–ductility synergy, for use in the high-performance engineering applications.

## Figures and Tables

**Table 1:** Chemical compositions of AXM080202 alloy (wt.%).

Alloy Composition	Al	Ca	Mn	Mg
Designed content	0.80	0.2	0.20	Bal.
Actual content (Mean ± SD)	0.82 ± 0.07	0.18 ± 0.03	0.22 ± 0.05	Bal.

**Table 2:** The mechanical properties of the aging-treatment AXM alloy sheet at different DCT durations, including yield tensile strength (TYS), ultimate tensile strength (UTS), elongation-to-failure (ε_f_), Vickers hardness (HV).

	TYS (MPa)	UTS (MPa)	εf (%)	HV
SAT	230 ± 5.0	290 ± 4.0	26.0 ± 1.0	72.8 ± 0.8
SCAT-0.5 h	250 ± 3.5	320 ± 4.0	28.2 ± 0.9	73.5 ± 0.6
SCAT-1 h	272 ± 2.5	332 ± 6.0	27.7 ± 0.5	75.4 ± 1.0
SCAT-12 h	280 ± 4.0	343 ± 5.0	27.3 ± 1.2	79.0 ± 1.0
SCAT-24 h	285 ± 2.5	345 ± 3.0	25.8 ± 0.8	78.5 ± 0.7
SCAT-48 h	288 ± 4.5	348 ± 4.5	25.2 ± 0.5	79.5 ± 0.5

## Data Availability

The original contributions presented in this study are included in the article. Further inquiries can be directed at the corresponding author.
